# Gender-specific associations among neck circumference, the rs9939609 *FTO* gene polymorphism, and the 14-year risk of metabolic syndrome in the Korean adult population

**DOI:** 10.4178/epih.e2024072

**Published:** 2024-08-23

**Authors:** Inkyung Baik

**Affiliations:** Department of Foods and Nutrition, College of Science and Technology, Kookmin University, Seoul, Korea

**Keywords:** Neck, Metabolic syndrome, Incidence, *FTO*, Waist circumference

## Abstract

**OBJECTIVES:**

Limited data exist on the relation between neck circumference (NC) and the risk of developing metabolic syndrome (MS). This study investigated gender-specific associations between NC and the 14-year risk of MS and explored the impact of the *FTO* rs9939609 polymorphism on these associations.

**METHODS:**

This population-based prospective cohort study involved 2,666 participants (1,301 men and 1,365 women), who were free of MS at baseline (2005-2006). Incident MS cases, defined by the presence of 3 or more criteria regarding blood pressure and blood levels of glucose, triglycerides, and high-density lipoprotein cholesterol, were identified through biennial examinations until 2020. NC measurements taken at baseline and between 2013 and 2014 were analyzed using Cox proportional hazard regression to determine gender-specific associations with MS risk.

**RESULTS:**

Controlling for potential confounders such as waist circumference (WC), significant associations were observed in both genders. Individuals in the highest NC quartile exhibited more than a 2-fold higher MS risk than those in the lowest quartile; with hazard ratios of 2.37 (95% confidence interval [CI], 1.74 to 3.22) for men and 2.65 (95% CI, 1.89 to 3.72) for women (p for trend <0.001). No significant interaction was found between the *FTO* polymorphism and NC. In diagnostic test analyses, NC and WC demonstrated comparable area under the curve values in both genders.

**CONCLUSIONS:**

The findings suggest that NC is as effective as WC for predicting the incidence of MS.

## GRAPHICAL ABSTRACT

[Fig f2-epih-46-e2024072]

## Key Message

Waist circumference has served as an anthropometric component of metabolic syndrome based on data regarding close associations with high blood pressure, hyperglycemia, and dyslipidemia. However, measurement errors of waist circumference due to the subject’s clothing, posture, fasting or postprandial state, and respiratory phase have been pointed out. This 14-year prospective cohort study including Korean adults has revealed that neck circumference can serve as a comparative indicator to waist circumference for predicting metabolic syndrome risk.

## INTRODUCTION

Although waist circumference (WC) is known as a simple and reliable measure of obesity and a component of metabolic syndrome (MS), potential technical errors in WC measurement can arise due to factors such as the subject’s clothing, posture, fasting or postprandial state, and respiratory phase [1]. In contrast, these errors can be minimized when measuring neck circumference (NC). Emerging evidence suggests that NC could be a valuable anthropometric marker for screening obesity, high blood pressure (HBP), high fasting glucose (HFG) levels, and dyslipidemia [2]. Several cross-sectional studies have shown a significant association between NC and the prevalence of MS, proposing specific NC cut-off points for diagnosing MS across various ethnic groups [3-8]. The results from receiver operating characteristic (ROC) analysis in these studies indicate that the area under the curve (AUC) for NC ranges from 0.73 to 0.82 in men and from 0.69 to 0.82 in women. This suggests that NC is a reasonably effective discriminator, despite the variability in cut-off points [3-8]. Additionally, 2 longitudinal studies have explored the relationship between changes in NC and MS components [9,10]. One study found a significant association between changes in NC and alterations in certain blood lipid levels, specifically triglycerides and low-density lipoprotein (LDL) cholesterol, although no such association was observed with high-density lipoprotein (HDL) cholesterol [9]. Another study examined the links between 1-year changes in 3 anthropometric measures—body weight, NC, and WC—and glucose metabolism profiles in obese women [10]. It revealed that longitudinal changes in NC and WC were inversely related to changes in an insulin sensitivity index. However, these studies only explored observational associations and did not establish a causal link between NC and the risk of MS. Data on the temporal relationship between NC and MS incidence from prospective studies remain sparse.

The fat mass and obesity-associated gene (*FTO*) has been identified as a susceptibility locus for obesity [11] and related diseases [12-15]. In particular, the rs9939609 variant of *FTO* is positively associated with MS components, including HBP [12], hyperglycemia [13], and reduced levels of HDL cholesterol [14]. However, a prospective study found no significant link between the rs9939609 variant of *FTO* and the risk of MS in adults with a body mass index (BMI) below 29 kg/m^2^ [15], despite *FTO* being considered a potential gene influencing MS [16]. Additionally, another study reported no association between this variant and NC [17], underscoring the need for further research into this relationship. To date, the interactive effects between *FTO* polymorphisms, NC, and MS risk have not been investigated.

This prospective study aimed to examine gender-specific associations between NC and the 14-year risk of MS, while also assessing these associations according to the *FTO* rs9939609 alleles. Additionally, the study evaluated whether NC can predict the incidence of MS and compared its diagnostic efficacy to that of WC.

## MATERIALS AND METHODS

### Study population

This study utilized data from an ongoing prospective cohort, which is part of the Korean Genome Epidemiology Study. Detailed information about the study population, methodologies, and procedures for health examinations and genome analysis has been documented in previous reports [18,19]. In brief, Korean adults residing in Ansan were recruited and enrolled as cohort members. These participants attended the Korea University Ansan Hospital for a standardized questionnaire-based interview and comprehensive health examination, conducted by trained health professionals from June 18, 2001, to January 29, 2003. Follow-up assessments, including similar interviews and health examinations, have been conducted biennially. Because information on both NC measures and total energy intake, identified as a significant confounding variable in a previous study [6], was available in the data collected between 2005 and 2006, this period was defined as the baseline for the current study. Accordingly, MS incidence cases were identified biennially during the follow-up period from 2007 to 2020. From the initial pool of 3,540 participants identified in the baseline period, prevalent MS cases and individuals lacking data on NC, WC, total energy intake, and the *FTO* rs9939609 polymorphism were excluded. Finally, data from 2,666 participants were utilized in the analysis.

### Definition of metabolic syndrome

MS was defined by the presence of 3 or more of the following 4 criteria: HBP, defined as a systolic/diastolic blood pressure (BP) ≥ 130/85 mmHg or the use of antihypertensive medications; HFG, defined as a fasting glucose level of ≥ 100 mg/dL or the use of glucose-lowering medications; high triglycerides (HTG), defined as ≥ 150 mg/dL or the use of medications for dyslipidemia; and low HDL cholesterol, defined as < 40 mg/dL for men and < 50 mg/dL for women [20]. WC was not included as a component of MS because it is closely related to NC. Therefore, WC and NC were analyzed together to assess their diagnostic capabilities in this study. New incident cases of MS over a 14-year period from 2007 to 2020 were analyzed in the current study.

### Anthropometric measurements

The primary exposure in this study was NC, which was measured while the participant stood in a relaxed position, with their head aligned in the Frankfort horizontal plane. A flexible tape measure, accurate to the nearest 0.1 cm, was positioned just below the laryngeal prominence and perpendicular to the long axis of the neck. Additionally, WC was measured at the narrowest point between the lower rib and the iliac crest, also to the nearest 0.1 cm. The average of 3 repeated measurements was recorded.

### Health examination and questionnaire

Periodic health examinations and questionnaire-based interviews were conducted. BP was measured using an electronic sphygmomanometer while participants were seated, following at least 5 minutes of rest. BP measurements were repeated after a minimum interval of 1 minute and recorded to the nearest 2 mmHg. The average of these measurements was calculated for both systolic and diastolic BP.

The questionnaire gathered data on socio-demographic characteristics, smoking status, alcohol consumption, sleep patterns, physical activity, and dietary intake. Dietary information, in particular, was collected using a semi-quantitative food frequency questionnaire developed by the Korea Disease Control and Prevention. Information on the methods used to calculate average daily alcohol consumption (g/day), average daily nutrient intake, and total metabolic equivalent scores (MET-hr/day) for physical activity can be found in another source [18].

### Genetic and biochemical analyses

Details on DNA sample handling, genotyping procedures, and quality control methods for single nucleotide polymorphism (SNP) genotype data have been documented in a previous study [21]. Briefly, genomic DNA was extracted from whole blood samples of 9,993 cohort members using the Affymetrix Genome-wide Human SNP Array 5.0 (Affymetrix Inc., Santa Clara, CA, USA). Data from 8,842 individuals, encompassing 500,568 SNPs, met the quality standards. These standards included call rates of at least 96%, no gender discrepancies, and average pairwise identity-by-state values not exceeding 0.8. From this SNP data, genotype information for the *FTO* rs9939609, which conformed to the Hardy-Weinberg equilibrium (p-value= 0.31), was extracted. Following a prior study [15], the TA and AA genotypes were identified as risk alleles, while the TT genotype was classified as non-risk. Biochemical assays for plasma glucose, serum triglycerides, and HDL cholesterol were conducted by a commercial laboratory. These were determined enzymatically through a colorimetric method using the Cobas analyzer (Roche Diagnostics, Mannheim, Germany).

### Statistical analysis

Descriptive statistics for the baseline characteristics of study participants were calculated based on gender-specific quartiles of NC. The chi-square test was used for categorical variables, and analysis of variance (ANOVA) was applied to continuous variables across the NC quartiles. Additionally, p-values for a linear trend were computed using the Cochran-Armitage test for trend and the ANOVA trend analysis, employing contrast coefficients.

To investigate the associations between NC quartiles and MS risk, Cox proportional hazards regression analysis was employed. This analysis generated hazard ratios (HRs) and 95% confidence intervals (CIs). The calculation of person-years extended from the baseline examination date to the earlier of either the date of death or the last follow-up date (December 31, 2020). Individuals who were lost to follow-up or died were censored in the analysis. The multivariable models incorporated several covariates: age (continuous), gender, WC (continuous), *FTO* rs9939609 allele status (risk or non-risk alleles), income level (≤ 2× 10^6^ or > 2× 10^6^ Korean won monthly), occupational type (office worker or non-office worker and other status), smoking status (never, former, or current smoker; < 10 and ≥ 10 cigarette/yr), alcohol consumption (abstainers or current consumers; < 15.1, 15.1-30.0, > 30.0 g/day), presence of sleep apnea episodes (yes or no), physical activity (quartiles of MET-hr/day), and total energy intake (quartiles). The assumption of proportionality for the multivariable models was tested and met. In the model, MS incidence cases, identified in the biennial follow-up examinations, served as the dependent variable, while baseline information (collected during 2005-2006) on NC, WC, and confounding variables was used during the followup period between 2007 and 2012. Updated information (collected during 2013-2014), except for physical activity and energy intake, was used as predictors of independent variables during the follow-up period between 2013 and 2020. Because survey questions on physical activity changed and dietary information was unavailable in the 2013-2014 follow-up data, only the baseline data for physical activity and energy intake was used. Significant predictors were identified through stepwise multivariable regression analysis, utilizing the SLENTRY and SLSTAY options to determine the best-fit model. Furthermore, ROC analysis was performed to assess whether NC can serve as a comparative indicator to WC for predicting incident MS cases. This involved comparing the AUC values derived from logistic regression models for NC and WC. Additionally, the associations between NC and MS risk stratified by the *FTO* rs9939609 allele variations (non-risk allele TT and risk alleles TA and AA) were analyzed. An interaction term between NC and these alleles was evaluated in the multivariable model.

All statistical analyses were performed using the SAS version 9.4 (SAS Institute Inc., Cary, NC, USA).

### Ethics statement

At both baseline and during follow-up visits, participants provided informed consent, which was approved by the institutional review board (IRB) of the study site. Additionally, the current study received approval from the IRB of Kookmin University (KMU-202312-HR-384) to obtain data collected from 2001 to 2020 by the Korea National Institute of Health (KNIH).

## RESULTS

Over a 14-year follow-up period (median: 11.7 years), 492 new cases of MS (247 men and 245 women) were identified. Table 1 presents the characteristics of the study participants, categorized by gender-specific quartiles of NC. The median values (range) for these quartiles were 35.6 cm (30.5-36.4), 37.0 cm (36.5-37.5), 38.3 cm (37.6-38.9), and 39.9 cm (39.0-45.5) for men, and 31.0 cm (28.4-31.7), 32.3 cm (31.8-32.8), 33.4 cm (32.9-34.0), and 35.0 cm (34.1-43.3) for women. Individuals with larger NC were more likely to carry the *FTO* rs9939609 A-risk alleles, to be office workers, and to report episodes of sleep apnea. WC and total energy intake increased with escalating NC quartiles. These characteristics were similarly observed in the results of separate analyses for men and women (Supplementary Materials 1 and 2).

Table 2 presents the association between NC quartiles and the incidence of MS. Although NC showed a strong correlation with WC (correlation coefficient: 0.61), there was no evidence of multicollinearity in the multivariable models. In these models, which controlled for age, WC, and other potential confounders, a significant association was observed across all participants, as well as separately among men and women (p for trend < 0.001). Compared to the lowest quartile, the highest NC quartile was linked to a 2-fold increase in the risk of MS for both genders. The multivariable HRs for the 4th quartile were 2.37 (95% CI, 1.74 to 3.22) for men and 2.65 (95% CI, 1.89 to 3.72) for women, relative to the gender-specific reference.

Table 3 details the results from the stepwise variable selection process, examining the association between various factors and the risk of MS. NC, WC, and age were identified as significant predictors of MS risk for both genders. For all participants, the multivariable HRs for a 1 cm increase in NC and WC were 1.08 (95% CI, 1.05 to 1.10) and 1.03 (95% CI, 1.02 to 1.04), respectively. Additionally, smoking and alcohol consumption were significant predictors for both the entire cohort and for men participants specifically. The *FTO* rs9939609 polymorphism and office work status also emerged as significant factors for men, whereas total energy intake was significant for women.

**Figure 1.**
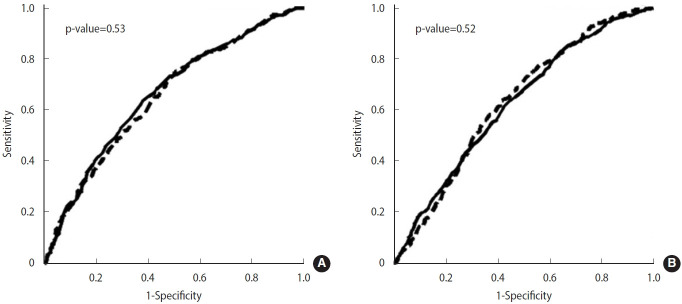
Results of receiver operating characteristic analysis for the associations of metabolic syndrome risk with neck circumference (NC) and waist circumference (WC) in women (A) and men (B). The curves are plotted with sensitivity (y-value) and [1-specificity] (x-value). Solid lines represent the curves for NC and dotted lines those for WC. The p-values for differences between the curve for WC and NC are indicated.

Figure 1 illustrates the gender-specific results of ROC analysis used to determine AUC values for the associations of NC and WC with the incidence of MS. For women, the AUC values were 0.66 for NC and 0.65 for WC, as shown in Figure 1A, with no significant differences between these values. For men, both NC and WC exhibited AUC values of 0.63, as depicted in Figure 1B, with no significant differences observed. The optimal cut-off points for NC, defined as the minimum distance from the upper left corner to the point on the ROC curve, were established at 38 cm for men and 33 cm for women.

Table 4 presents the results of a joint analysis that examines the association between NC, the *FTO* rs9939609 alleles, and the risk of MS. Participants with a larger NC were found to have a higher risk of MS compared to those with a narrower NC, regardless of their allele type. This pattern was consistent across all groups, including both men and women. Although individuals carrying the A-risk alleles exhibited a slightly higher risk of MS than those with non-risk alleles, the interactions between NC and the alleles were not statistically significant. Moreover, after adjusting for WC and other confounding variables, no association was found between NC and the *FTO* alleles.

## DISCUSSION

A 14-year prospective cohort study involving middle-aged and older Korean adults revealed a significant link between NC and the risk of MS, which is characterized by a combination of HBP, hyperglycemia, and dyslipidemia. NC was identified as a significant predictor of MS, with its predictive power comparable to that of WC in both men and women. In addition to NC and WC, other factors influencing the risk of MS included non-modifiable elements such as aging and genetic predisposition (specifically the presence of *FTO* rs9939609 A-risk alleles), as well as modifiable factors including smoking, heavy alcohol consumption, office work, and high caloric intake.

The utility of NC measurement extends beyond its role as a simple and convenient anthropometric tool; it also serves as a valuable indicator of MS prevalence. Multiple studies involving diverse ethnic groups have demonstrated a correlation between NC and the prevalence of MS and its components [2-8]. Some researchers have suggested that NC could be used as an alternative to WC as an anthropometric measure for identifying MS prevalence [7,8,22]. In a cross-sectional study of Chinese older adults, NC and WC were similarly associated with MS prevalence, with odds ratios of 1.2 for NC and 1.1 for WC among men, and 1.3 for NC and 1.1 for WC among women [22]. A study among younger African adults found no significant difference in the AUC values for NC and WC in men, while AUC values for WC were significantly higher than those for NC in women [4]. In the current study, no significant difference in the AUC values for NC and WC was observed in both genders, and the magnitude of the association between NC and MS risk was similar for both men and women. The discrepancies between the results of the previous study [4] and the current study may be partly due to differences in outcomes and the age ranges of the participants. The optimal NC cut-off points identified in Asian populations ranged from 37 cm to 40 cm for men and from 33 cm to 35 cm for women [23]. In 2 Korean cross-sectional studies [6,24], the optimal NC cut-off points were observed at 38 cm for men and 33 cm for women. However, data on the relationship between NC and MS risk, as well as the optimal NC cut-off points for predicting incident MS cases, remain limited. The current study found that NC was significantly associated with the 14-year risk of MS, independent of WC, in both men and women. The predictive ability of NC for MS incidence was comparable to that of WC. Additionally, this study supports the use of previously identified optimal NC cut-off points for predicting MS incident cases, despite the anthropometric changes observed during the extensive follow-up period. However, the AUC values for NC and WC were not as high as those reported in an earlier study [6].

An earlier study that analyzed data from the same cohort identified a significant association between the *FTO* rs9939609 alleles and a 6-year risk of MS in participants with a BMI of 29 kg/m^2^ or higher. However, no such association was found in individuals with a BMI below 29 kg/m^2^ [15]. In contrast, the current study, which utilized data from a longer follow-up period, found a significant association between this *FTO* variant and the risk of MS in men, regardless of their BMI. Consistent with previous findings [17], the current study also found no association between the *FTO* rs9939609 alleles and NC.

The *FTO* gene encodes a 2-oxoglutarate-dependent nucleic acid demethylase that plays a role in energy homeostasis [25], regulation of food intake [26], and control of body fat content [27]. Numerous population-based genetic studies have associated *FTO* with obesity [11] and traits related to obesity [12-15].

The current study observed that smoking and heavy alcohol consumption were associated with an increased risk of MS, particularly among men. This finding is consistent with previous research [15]. Meta-analysis studies have also shown that these behaviors exacerbate components of MS, including WC, HBP, HFG, and HTG [28,29]. Additionally, the study found that office work increased the risk of MS compared to non-office work settings among men. This association may be attributed to factors such as low levels of physical activity, psychological distress from confined workspaces, and exposure to indoor air pollutants [30]. The significant associations with smoking, heavy alcohol consumption, and occupational status observed exclusively in men may be due to the higher prevalence of these risk factors in this group. Conversely, the study identified a significant association between total energy intake and a 14-year risk of MS exclusively in women, whereas a previous study using the same cohort found no such association in either gender [15].

This study’s strength lies in the consistent measurements of NC at baseline and during follow-up by trained researchers, ensuring that updated measurements were incorporated into the analysis. However, a limitation of the study is that the incidence of MS was not identified in 18% of participants due to loss to follow-up. Despite this, the distribution of NC quartiles was similar between participants lost to follow-up and those whose outcomes were identified. In terms of anthropometric measurements, a single measurement of NC and 3 repeated measurements of WC were obtained. Although these measurements were conducted by a trained researcher following the KNIH’s standardized protocol, potential measurement errors cannot be completely ruled out. Another limitation is the inability to update 2 variables, total energy intake and physical activity, which may have introduced residual confounding. Additionally, the applicability of the findings is limited to Asian adults within similar age ranges.

The study findings have implications for both clinical and public health practices. NC may serve as an alternative to WC for predicting the risk of MS. Furthermore, modifications in lifestyle factors such as smoking, alcohol consumption, and diet are recommended to prevent MS.

## Figures and Tables

**Figure f2-epih-46-e2024072:**
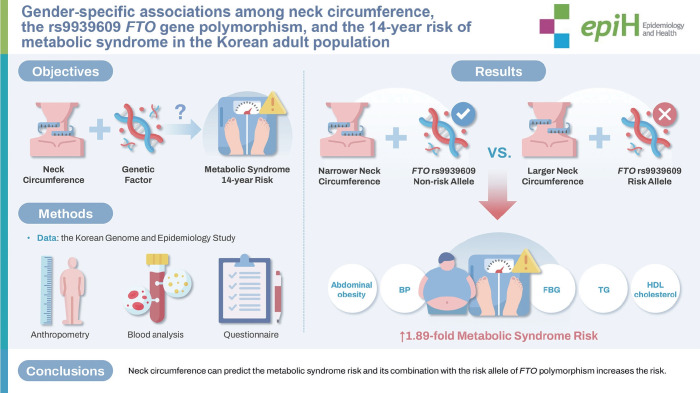


**Table 1.** Baseline characteristics of study participants according to the gender-specific quartiles of NC (n=2,666)

Characteristics	Quartiles of NC (median, cm)				p for trend
	1st (31.7)	2nd (32.8)	3rd (34.0)	4th (39.0)	
No. of participants (% of total)	671 (25.2)	658 (24.7)	682 (25.6)	655 (24.6)	
Age (yr)	52.0±7.4	51.8±7.1	52.2±7.1	52.6±7.6	0.11
Men	48.7	48.3	46.9	51.3	0.48
*FTO* rs9939609 minor alleles^1^	20.0	21.3	25.1	26.6	<0.01
Waist circumference (cm)	74.1±5.6	78.6±5.6	82.1±5.7	87.2±6.6	<0.001
Low income^2^	33.8	32.8	31.4	32.2	0.43
Office worker	24.9	27.1	31.5	30.5	<0.01
Current smokers	14.8	19.5	15.5	16.3	0.89
Current alcohol drinkers	47.1	48.8	51.8	51.2	0.08
Having sleep apnea episodes	8.8	10.6	17.0	19.5	<0.001
Physical activity (MET-hr/day)^3^	45.5±7.8	44.9±8.1	44.9±7.3	44.7±7.4	0.07
Total energy intake (kcal/day)	1,798±500	1,848±511	1,860±514	1,922±556	<0.001

Values are presented as mean±standard deviation or %. NC, neck circumference; MET, total metabolic equivalent.

1 Genotypes TA and AA.

2 Average monthly wage <2×10^6^ Korean won.

3 MET was calculated for daily physical activity.

**Table 2.** Hazard ratios for the association between gender-specific quartiles of NC and the 14-year incidence of metabolic syndrome

Variables	Quartiles of NC	No. of cases/PY	Adjusted		
			Age	Age and WC	Multivariable^1^
All (n=2,666)	1st	159/7,110	1.00 (reference)	1.00 (reference)	1.00 (reference)
	2nd	236/6,200	1.71 (1.40, 2.09)	1.51 (1.23, 1.85)	1.53 (1.24, 1.88)
	3rd	306/5,886	2.34 (1.94, 2.84)	1.88 (1.53, 2.31)	1.99 (1.61, 2.45)
	4th	376/5,390	3.09 (2.56, 3.72)	2.16 (1.73, 2.69)	2.50 (2.00, 3.14)
	p for trend		<0.001	<0.001	<0.001
Men (n=1,301)	1st	90/3,137	1.00 (reference)	1.00 (reference)	1.00 (reference)
	2nd	151/2,723	1.95 (1.50, 2.53)	1.81 (1.38, 2.36)	1.69 (1.29, 2.21)
	3rd	175/2,484	2.50 (1.94, 3.22)	2.18 (1.64, 2.89)	2.02 (1.51, 2.68)
	4th	197/2,461	2.84 (2.21, 3.64)	2.33 (1.72, 3.17)	2.37 (1.74, 3.22)
	p for trend		<0.001	<0.001	<0.001
Women (n=1,365)	1st	69/3,973	1.00 (reference)	1.00 (reference)	1.00 (reference)
	2nd	85/3,477	1.37 (0.99, 1.89)	1.29 (0.93, 1.78)	1.26 (0.91, 1.74)
	3rd	131/3,402	2.10 (1.57, 2.81)	1.88 (1.38, 2.57)	1.84 (1.34, 2.51)
	4th	179/2,929	3.15 (2.38, 4.16)	2.61 (1.86, 3.65)	2.65 (1.89, 3.72)
	p for trend		<0.001	<0.001	<0.001

Values are presented as hazard ratio (95% confidence interval). NC, neck circumference; WC, waist circumference; PY, person-years: MET, total metabolic equivalent.

1 Data are adjusted for age (continuous), gender, WC (continuous), *FTO* rs9939609 allele status (risk or non-risk alleles), income level (<2×10^6^ or ≥2×10^6^ Korean won monthly), occupational type (office worker or non-office worker and other status), smoking status (never, former, or current smoker; <10 and ≥10 cigarette/yr), alcohol consumption (abstainers, current consumers; <15.1, 15.1-30.0, >30.0 g/day), presence of sleep apnea episodes (yes or no), physical activity (quartiles of MET-hr/day), and total energy intake (quartiles).

**Table 3.** Stepwise selection of variables associated with the 14-year incidence of metabolic syndrome

Variables	Selected variables	Unit or reference	p-value	Multivariable^1^
All (n=2,666)	Neck circumference	1 cm	<0.001	1.08 (1.05, 1.10)
	Age	1 yr	<0.001	1.03 (1.02, 1.04)
	Waist circumference	1 cm	<0.001	1.03 (1.02, 1.04)
	Smoking	Non-smoking	<0.001	1.37 (1.17, 1.62)
	Heavy alcohol consumption	Non-drinking and light to moderate alcohol consumption	<0.010	1.31 (1.08, 1.58)
Men (n=1,301)	Neck circumference	1cm	<0.001	1.08 (1.03, 1.12)
	Waist circumference	1cm	<0.001	1.03 (1.01, 1.04)
	Smoking	Non-smoking	<0.001	1.37 (1.15, 1.63)
	Age	1 yr	<0.010	1.02 (1.01, 1.03)
	Office worker	Non-office worker and other status	<0.050	1.23 (1.04, 1.47)
	Heavy alcohol consumption	Non-drinking and light to moderate alcohol consumption	<0.050	1.23 (1.01, 1.51)
	*FTO* rs9939609 risk alleles	Non-risk allele	<0.050	1.21 (1.00, 1.45)
Women (n=1,365)	Age	1 yr	<0.001	1.06 (1.04, 1.07)
	Neck circumference	1 cm	<0.001	1.09 (1.05, 1.13)
	Waist circumference	1 cm	<0.001	1.03 (1.02, 1.04)
	Total energy intake	500 kcal	<0.050	1.12 (1.01, 1.23)

Values are presented as hazard ratio (95% confidence interval). MET, total metabolic equivalent.

1 Data are adjusted for age (continuous), gender, waist circumference (continuous), *FTO* rs9939609 allele status (risk or non-risk alleles), income level (<2×10^6^ or ≥2×10^6^ Korean won monthly), occupational type (office worker or non-office worker and other status), smoking status (never, former, or current smoker; <10 and ≥10 cigarette/yr), alcohol consumption (abstainers, current consumers; <15.1, 15.1-30.0, >30 g/day), presence of sleep apnea episodes (yes or no), physical activity (quartiles of MET-hr/day), and total energy intake (quartiles).

**Table 4.** Association between gender-specific quartiles of NC and the 14-year incidence of metabolic syndrome

Variables	Groups of NC (quartile)	*FTO* rs9939609 alleles^1^	aHR (95% CI)			p for interaction
			Age	Age and WC	Multivariable^2^	
All (n=2,666)	1st and 2nd	Non-risk allele	1.00 (reference)	1.00 (reference)	1.00 (reference)	0.23
	1st and 2nd	Risk alleles	1.14 (0.90, 1.44)	1.11 (0.88, 1.39)	1.11 (0.88, 1.40)	
	3rd and 4th	Non-risk allele	2.04 (1.77, 2.35)	1.54 (1.31, 1.81)	1.65 (1.40, 1.94)	
	3rd and 4th	Risk alleles	2.25 (1.87, 2.71)	1.70 (1.39, 2.07)	1.89 (1.54, 2.31)	
Men (n=1,301)	1st and 2nd	Non-risk allele	1.00 (reference)	1.00 (reference)	1.00 (reference)	0.57
	1st and 2nd	Risk alleles	1.20 (0.90, 1.60)	1.20 (0.90, 1.60)	1.24 (0.93, 1.66)	
	3rd and 4th	Non-risk allele	1.88 (1.55, 2.27)	1.52 (1.23, 1.90)	1.53 (1.23, 1.91)	
	3rd and 4th	Risk alleles	2.14 (1.66, 2.75)	1.78 (1.36, 2.33)	1.82 (1.39, 2.39)	
Women (n=1,365)	1st and 2nd	Non-risk allele	1.00 (reference)	1.00 (reference)	1.00 (reference)	0.22
	1st and 2nd	Risk alleles	0.92 (0.62, 1.37)	0.90 (0.60, 1.33)	0.87 (0.58, 1.29)	
	3rd and 4th	Non-risk allele	2.11 (1.69, 2.63)	1.76 (1.38, 2.25)	1.75 (1.37, 2.25)	
	3rd and 4th	Risk alleles	2.32 (1.76, 3.07)	1.88 (1.38, 2.55)	1.87 (1.37, 2.56)	

NC, neck circumference; aHR, adjusted hazard ratio; CI, confidence interval; WC, waist circumference; MET, total metabolic equivalent.

1 TT is the non-risk allele, while TA and AA are risk alleles.

2 Data are adjusted for age (continuous), gender, waist circumference (continuous), income level (<2×10^6^ or ≥2×10^6^ Korean won monthly), occupational type (office worker or non-office worker and other status), smoking status (never, former, or current smoker; <10 and ≥10 cigarette/yr), alcohol consumption (abstainers, current consumers; <15.1, 15.1-30.0, >30.0 g/day), presence of sleep apnea episodes (yes or no), physical activity (quartiles of MET-hr/day), and total energy intake (quartiles).

## Supplementary materials

Supplementary Material 1. Baseline characteristics of 1301 men according to the quartiles of neck circumference

Supplementary Material 2. Baseline characteristics of 1365 women according to the quartiles of neck circumference
