# Supplementary material for: Gender-specific associations among neck circumference, the rs9939609 FTO gene polymorphism, and the 14-year risk of metabolic syndrome in the Korean adult population
Source: Epidemiol Health. 2024 Aug 23;46:e2024072. doi: 10.4178/epih.e2024072 (PMC11826040; doi:10.4178/epih.e2024072)
Supplement: Supplementary Material 1. — Baseline characteristics of 1301 men according to the quartiles of neck circumference [file epih-46-e2024072-Supplementary-1.docx]

**Supplementary Material 1**. Baseline characteristics of 1301 men according to the quartiles of neck circumference

|  | Quartiles of neck circumference (median, cm) | | | | p-value |
| --- | --- | --- | --- | --- | --- |
| Characteristics | 1st quartile (35.6) | 2nd quartile (37.0) | 3rd quartile (38.3) | 4th quartile (39.9) | for trend |
| Number of participants (% of total) | 327 (25.1) | 318 (24.4) | 320 (24.6) | 336 (25.8) |  |
| Age, years | 52.9±7.5 | 51.8±7.0 | 51.8±7.1 | 51.4±6.7 | <0.01 |
| *FTO* rs9939609 minor alleles***^1)^***, % | 22.9 | 21.4 | 24.4 | 25.6 | 0.29 |
| Waist circumference, cm | 76.7±5.5 | 81.2±4.7 | 84.5±4.8 | 88.7±5.5 | <0.001 |
| Low income***^2)^***, % | 31.8 | 28.3 | 22.8 | 22.0 | <0.01 |
| Office worker, % | 37.6 | 42.5 | 52.2 | 46.4 | <0.01 |
| Current smokers, % | 28.4 | 37.7 | 31.9 | 30.7 | 0.94 |
| Current alcohol drinkers, % | 68.2 | 70.1 | 77.8 | 73.2 | <0.05 |
| Having sleep apnea episodes, % | 14.1 | 17.3 | 28.1 | 29.8 | <0.001 |
| Physical activity***^3)^***, MET-hours/d | 46.5±9.7 | 45.8±9.5 | 45.2±8.0 | 45.3±8.2 | 0.06 |
| Total energy intake, kcal/d | 1922±484 | 1997±513 | 2040±499 | 2087±577 | <0.001 |

Mean ± standard deviation or proportions in the cell

***^1)^*** genotype TA and AA

***^2)^*** Average monthly wage < 2×10^6^ won

***^3)^*** Total metabolic equivalent was calculated for daily physical activity
