# Supplementary material for: Gender-specific associations among neck circumference, the rs9939609 FTO gene polymorphism, and the 14-year risk of metabolic syndrome in the Korean adult population
Source: Epidemiol Health. 2024 Aug 23;46:e2024072. doi: 10.4178/epih.e2024072 (PMC11826040; doi:10.4178/epih.e2024072)
Supplement: Supplementary Material 2. — Baseline characteristics of 1365 women according to the quartiles of neck circumference [file epih-46-e2024072-Supplementary-2.docx]

**Supplementary Material 2**. Baseline characteristics of 1365 women according to the quartiles of neck circumference

|  | Quartiles of neck circumference (median, cm) | | | | p-value |
| --- | --- | --- | --- | --- | --- |
| Characteristics | 1st quartile (31.0) | 2nd quartile (32.3) | 3rd quartile (33.4) | 4th quartile (35.0) | for trend |
| Number of participants (% of total) | 344 (25.2) | 340 (24.9) | 362 (26.5) | 319 (23.4) |  |
| Age, years | 51.3±7.2 | 51.8±7.1 | 52.6±7.2 | 53.8±8.3 | <0.001 |
| *FTO* rs9939609 minor alleles***^1)^***, % | 17.2 | 21.2 | 25.7 | 27.6 | <0.001 |
| Waist circumference, cm | 71.7±5.3 | 76.1±5.3 | 80.0±5.6 | 85.6±7.3 | <0.001 |
| Low income***^2)^***, % | 35.8 | 37.1 | 39.0 | 43.0 | 0.05 |
| Office worker, % | 12.8 | 12.7 | 13.3 | 13.8 | 0.66 |
| Current smokers, % | 1.74 | 2.35 | 1.10 | 1.25 | 0.37 |
| Current alcohol drinkers, % | 27.0 | 28.8 | 28.7 | 27.9 | 0.81 |
| Having sleep apnea episodes, % | 3.78 | 4.41 | 7.18 | 8.78 | <0.01 |
| Physical activity***^3)^***, MET-hours/d | 44.5±5.4 | 44.0±6.3 | 44.7±6.6 | 44.0±6.4 | 0.53 |
| Total energy intake, kcal/d | 1680±487 | 1708±469 | 1700±472 | 1749±476 | 0.09 |

Mean ± standard deviation or proportions in the cell

***^1)^*** genotype TA and AA

***^2)^*** Average monthly wage < 2×10^6^ won

***^3)^*** Total metabolic equivalent was calculated for daily physical activity
